# Successful management of testicular primitive neuroectodermal tumor with multiple bone metastases

**DOI:** 10.1002/iju5.12431

**Published:** 2022-03-26

**Authors:** Takuya Takasawa, Tomonori Minagawa, Takahisa Domen, Toshirou Fukushima, Yu Fujii, Mai Iwaya, Tomonobu Koizumi, Teruyuki Ogawa, Osamu Ishizuka

**Affiliations:** ^1^ Department of Urology Shinshu University School of Medicine Matsumoto Japan; ^2^ Department of Hematology and Medical Oncology Shinshu University School of Medicine Matsumoto Japan; ^3^ Department of Neurosurgery Shinshu University School of Medicine Matsumoto Japan; ^4^ Department of Laboratory Medicine Shinshu University Hospital Matsumoto Japan

**Keywords:** chemotherapy, germ cell tumor, malignant transformation of teratoma, primitive neuroectodermal tumor

## Abstract

**Introduction:**

Germ cell tumor with malignant transformation is extremely rare. We present a case of testicular primitive neuroectodermal tumor with multiple metastases that was effectively managed by surgery, irradiation, and second‐line chemotherapy.

**Case presentation:**

A 22‐year‐old man was diagnosed as having teratoma including primitive neuroectodermal tumor with lymph node and multiple bone metastases. Five months afterwards the first‐line therapy, his skull metastasis recurred. Vincristine, doxorubicin, and cyclophosphamide therapy followed by vincristine, actinomycin D, and cyclophosphamide therapy was given as second‐line chemotherapy. Computed tomography revealed no disease progression 3 months after the treatments.

**Conclusion:**

Metastatic primitive neuroectodermal tumor may be successfully managed by multidisciplinary cancer treatment.

**Abbreviations & Acronyms**
cPNET: central primitive neuroectodermal tumor
CT: computed tomography
GCT: germ cell tumor
PET: positron emission tomography
PNET: primitive neuroectodermal tumor
pPNET: peripheral primitive neuroectodermal tumor
RPLND: retroperitoneal lymph node dissection
VAC: vincristine, actinomycin D, and cyclophosphamide
VDC: vincristine, doxorubicin, and cyclophosphamide

**Keynote message**

A 22‐year‐old man was diagnosed as having testicular PNET with lymph node and multiple bone metastases. A left high inguinal orchiectomy, first‐line chemotherapy, and RPLND were given. Five months later, the skull metastasis had increased in size. Radiation of the metastatic lesions and VDC followed by VAC therapy was effective in managing the metastatic lesions.

## Introduction

Testicular GCT is the most common solid tumor diagnosed in young men.^1^ On the contrary, GCT with malignant transformation into somatic‐type malignancy is extremely rare,^1^, ^2^ in 1979–2011, 121 patients were reported and of which 112 were testicular primary.^1^ Among them, sarcoma and PNET are the most frequent histologies.^1^ Cisplatin‐based chemotherapy regimens have proven to be ineffective in the treatment of teratoma with malignant transformation, including PNET.^3^, ^4^ We herein describe a case of testicular PNET with para‐aortic lymph node and multiple bone metastases. Irradiation and second‐line chemotherapy were effective in successfully managing the metastatic lesions.

## Case presentation

**Fig. 1:**
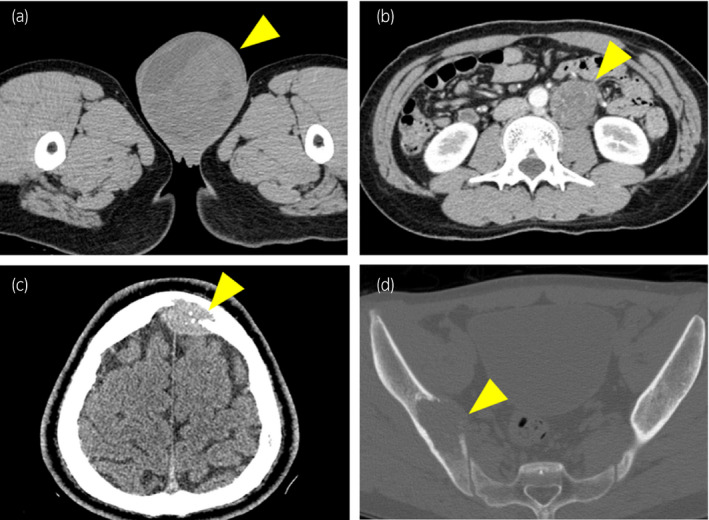
CT revealed a left testicular tumor and metastatic tumors. (a) A large left testicular tumor of 14 × 9 cm was observed in the testis (arrowhead). (b) An enlarged para‐aortic lymph node was evident (arrowhead). (c) A metastatic tumor with bone destruction was identified in the frontal bone (arrowhead). (d) An osteolytic tumor was observed in the left ilium (arrowhead).

**Fig. 2:**
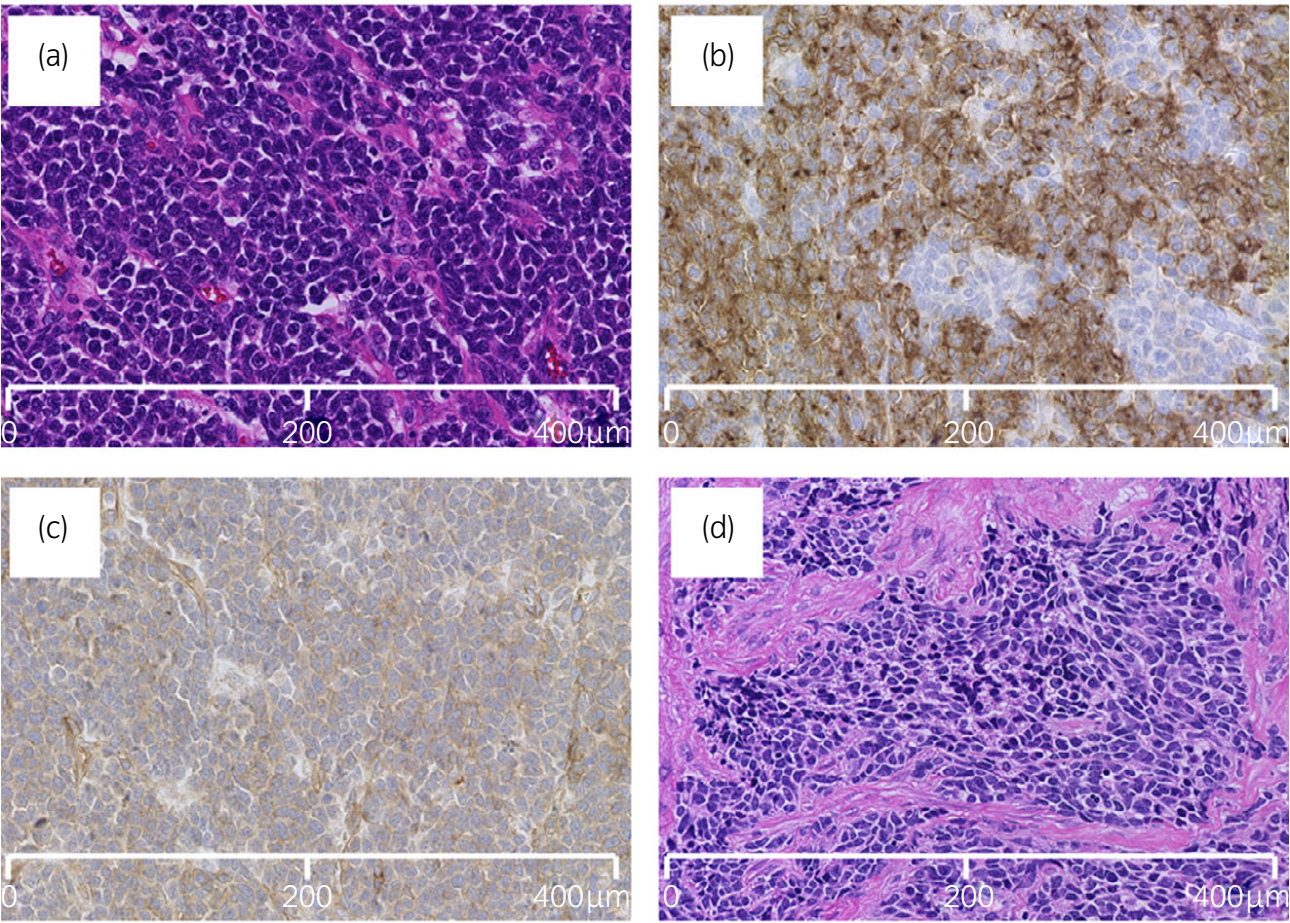
Histological findings of the primary lesion of the testis and the metastatic lesion of the skull. (a) In hematoxylin and eosin staining of the primary tumor, a sheet‐like cell sequence with highly chromatin‐stained round‐to‐polygonal nuclei was observed. (b) Immunohistochemically, the primary tumor was diffusely immunoreactive for synaptophysin, (c) and weakly focally for CD99 (MIC2 protein). (d) In hematoxylin and eosin staining of the skull metastasis, cells and sequence images similar to those of the primary tumor were observed.

**Fig. 3:**
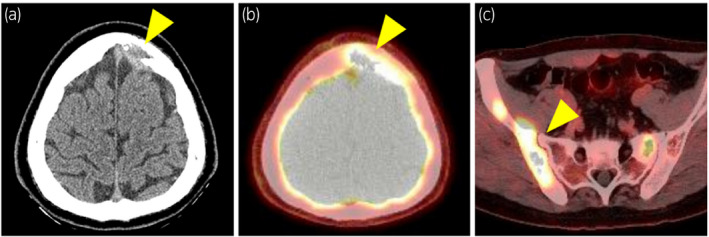
Imaging evaluation at tumor marker re‐elevation showed exacerbation of the bone metastatic lesions. (a) CT revealed progressive bone destruction of the frontal bone and an increase in soft tissue tumors (arrowhead). (b) PET showed high accumulation in the frontal bone lesions (arrowhead). (c) PET disclosed high accumulation in osteolytic lesions in the left iliac bone (arrowhead).

A 22‐year‐old man with a half‐year history of swelling of the left scrotum presented with anterior chest pain. CT revealed a large 14 × 9 cm mass in the left scrotum and metastatic lesions in the para‐aortic lymph node, skull, pelvic bones, sternum, and vertebrae (Fig. 1a–d). The tumor markers were elevated for alpha‐fetoprotein (513.4 ng/mL; normal range: <10.0 ng/mL), human chorionic gonadotropin (5.81 mIU/mL; normal range: <2.0 mIU/mL), neuron‐specific enolase (204 ng/mL; normal range: <12.0 ng/mL), and lactate dehydrogenase (842 U/L; normal range: 124–222 U/L). A left high inguinal orchiectomy was performed, which enabled the histological diagnosis of GCT of immature teratoma including PNET (Fig. 2a–c). Clinically,the patient was diagnosed as having non‐seminomatous GCT with para‐aortic lymph node metastasis and multiple bone metastases defined as pT2N3M1b Stage IIIC, and was given a poor prognosis according to the International Germ Cell Consensus Classification Grade.^5^ Four cycles of BEP (bleomycin 30 mg/body, etoposide 100 mg/m^2^, and cisplatin 20 mg/m^2^) therapy were given in combination with denosumab 120 mg every 4 weeks. After chemotherapy, tumor markers returned to normal range and the metastasis in the para‐aortic lymph node had decreased in size. Subsequent RPLND led to a pathological diagnosis of viable teratoma without PNET. One month afterwards, PET‐CT revealed strong accumulation in the sternum and pelvic bone. Since no additional metastases or enlargements in bone metastatic lesions were evident, he was followed closely under continued denosumab administration. Five months after RPLND, his serum lactate dehydrogenase and neuron‐specific enolase had increased to 260 U/L and 19.9 ng/mL, respectively. CT revealed soft tissue mass formation with skull destruction (Fig. 3a). As the skull tumor was very close to the sagittal sinus, only biopsy procedure was performed for histological evaluation instead of complete tumor resection and the lesion was diagnosed as metastatic PNET (Fig. 2d). PET‐CT also showed strong accumulation in the osteolytic bone metastases of the skull, sternum, and pelvic bone (Fig. 3b,c). External beam radiation was applied to the osteolytic metastatic PNET lesions (total dose of 39 Gy in 3 fractions for the skull and 30 Gy in 3 fractions for the pelvic bone) along with five courses of VDC (vincristine 2 mg, doxorubicin 75 mg/m^2^, and cyclophosphamide 1200 mg/m^2^) therapy. Afterwards, 12 courses of VAC (vincristine 2 mg, actinomycin D 2.5 mg, and cyclophosphamide 1200 mg/m^2^) therapy were added due to the limited dose of doxorubicin. CT revealed no disease progression 3 months after all 17 courses of VDC and VAC therapy and no other metastatic lesions. The patient is currently under close follow‐up with continued denosumab administration.

## Discussion

PNET is an immature malignant tumor derived from the neuroectoderm, and classified into two groups: cPNET originating from the central nervous system and pPNET deriving from other tissues. PNET from testicular GCT is predominantly classified as cPNET,^6^ however, pPNET derived from testicular germinoma has also been reported.^6^, ^7^ Since pPNET and Ewing's sarcoma share common neuroendocrine markers and genetic abnormalities, they have recently referred to as the pPNET/Ewing's sarcoma family.^7^ Although they commonly metastasize into the lymph nodes, bones, and lungs, to the best of our knowledge, metastatic lesion in the skull have not yet been described.

PNET can only be diagnosed by histopathological examination. Tumors exhibit small round cells with round‐to‐oval nuclei and may show rosette‐like arrangement.^6^, ^7^ In immunohistopathological examinations, both cPNET and pPNET often stain positively for such neuroendocrine markers as CD56, synaptophysin, and chromogranin A.^6^, ^7^ CD99 is frequently positive in pPNET but negative in cPNET, which is useful for distinguishing between the entities.^6^, ^7^

The treatment of PNET remains uncertain. Although cisplatin‐based chemotherapy regimens have good outcomes in non‐transformed GCT, they were found to be ineffective for PNET.^3^ On the contrary, several reports have shown that the chemotherapy for Ewing's sarcoma is also effective for PNET. Ehrlich *et al*. described that 8 of 10 PNET patients achieved an objective response to chemotherapy of VAC alternating with ifosfamide plus etoposide.^8^ Elsewhere, Angela *et al*. found that VAC therapy for CD99‐positive metastatic PNET malignantly transformed from testicular teratoma provided asymptomatic relief for 18 months.^3^

The presented case had a primary testicular tumor and metastatic lesions in the para‐aortic lymph node and multiple bones. Pathological examination of the primary lesion and a skull biopsy revealed teratoma including PNET. In immunohistopathological analysis, the neuroendocrine markers CD56, synaptophysin, and chromogranin A were all positive. As CD99 was weakly positive in the former examination and difficult to judge in the latter, the tumor could not be conclusively determined as pPNET. The first‐line chemotherapy was ineffective for PNET, as evidenced by the metastatic lesions. However, VDC followed by VAC therapy was able to control the metastatic lesions following the surgery, radiation, and first‐line chemotherapy. VDC following VAC therapy may therefore represent a therapeutic option for metastatic pPNET. However, careful follow‐up for metastatic lesions using CT and PET/CT remains essential. Further investigation is needed to establish successful testicular PNET treatment regimens.

## Author Contributions

Takuya Takasawa: Writing – original draft. Tomonori Minagawa: Writing – review & editing. Takahisa Domen: Resources. Toshirou Fukushima: Resources. Yu Fujii: Resources. Mai Iwaya: Resources. Tomonobu Koizumi: Resources. Teruyuki Ogawa: Resources. Osamu Ishizuka: Resources.

## Conflict of interest

The authors declare no conflict of interest.

## Approval of the research protocol by an Institutional Reviewer Board

Not Applicable.

## Informed consent

Written informed consent was obtained from patient for publication of this case report and accompanying images.

## Registry and the Registration No. of the study/trial

Not Applicable.
